# Supplementary material for: Insulin-Degrading Enzyme Regulates mRNA Processing and May Interact with the CCR4-NOT Complex
Source: Cells. 2025 May 28;14(11):792. doi: 10.3390/cells14110792 (PMC12153694; doi:10.3390/cells14110792)
Supplement: Supplementary file 1 [file cells-14-00792-s001.zip › cells-3476596-supplementary.pdf]

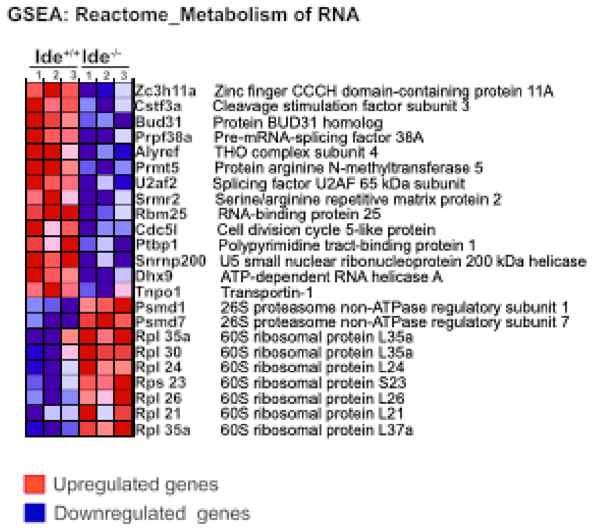

Figure S1. Upregulation of proteins related to RNA metabolism in NOD *Ide*$^{+/+}$ islets. The heatmap depicts differential expression of proteins from *Ide*$^{+/+}$ and *Ide*$^{-/-}$ NOD islet cells in the curated Reactome RNA metabolism pathway (GSEA analysis).

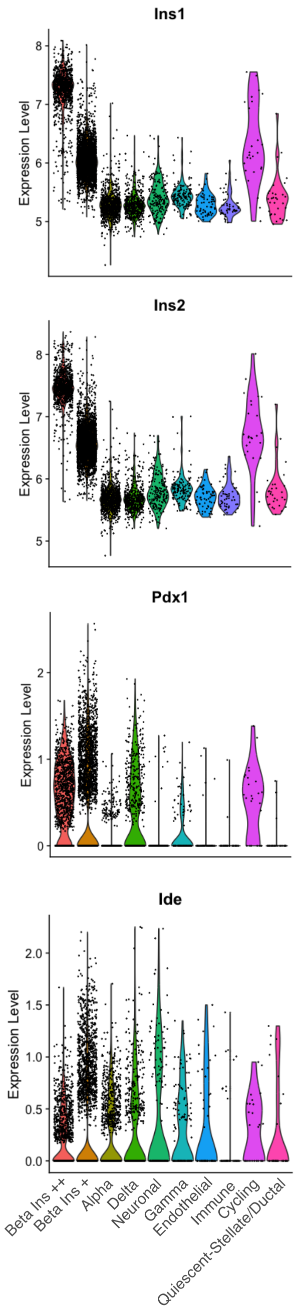

Figure S2. *Ide* expression correlates with intermediate insulin expression. Violin plots show the expression level of the *Ins1*, *Ins2*, *Pdx1* and *Ide* genes in selected populations among *Ide*$^{+/+}$ C57BL6 islet cells.

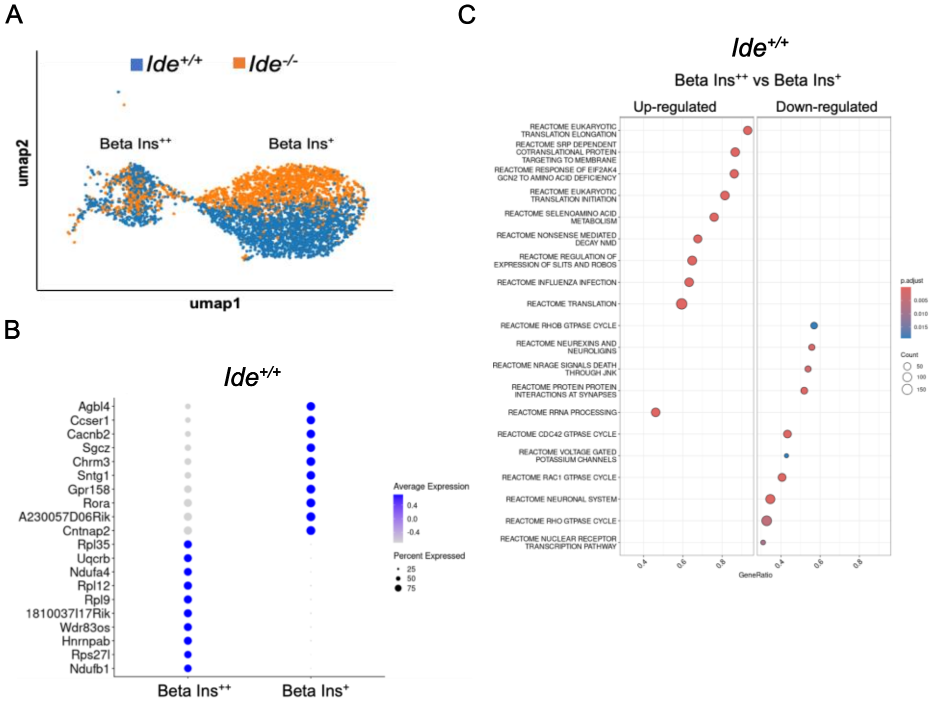

Figure S3. Single cell RNA-seq analysis of beta cell subsets. a) Manifold Approximation and Projection plot (UMAP) depicting clusters of beta cells expressing different *Ins* mRNA levels in the two genotypes. The color-coded indicates the genotype. b) Dot plots show the genes with the highest expression changes in *Ins*$^{++}$ and *Ins*$^{+}$ beta cells. c) Pathways significantly associated with upregulated or downregulated genes in *Ins*$^{+/+}$ compared to *Ins*$^{+}$ beta cells (FDR < 0.05) were identified with Gene set enrichment analysis (GSEA), using the Molecular Signatures Database (MSigDBr) Reactome gene sets. Gene Ratio indicates the proportion of up- or downregulated genes among each gene set, and Count depicts the number of up- or downregulated genes in the set.

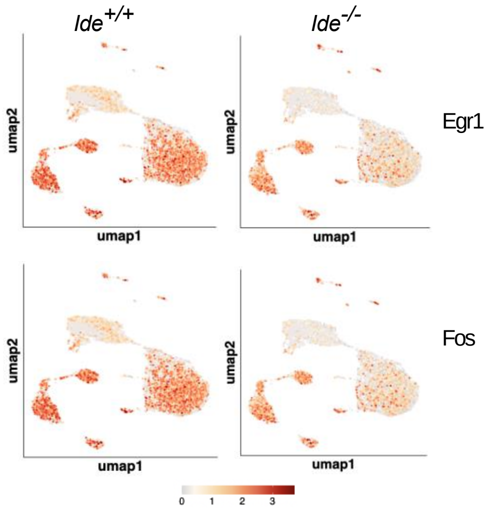

Figure S4. *Ide* expression correlates with *Egr1* and *Fos* expression. Egr1 and Fos expression is downregulated in *Ide*$^{-/-}$ beta and alpha cells and in *Ide*$^{+/+}$ beta cells with high insulin expression. UMAPs depict clusters of cells expressing *Egr1* (top panels) and *Fos* (bottom panels) from *Ide*$^{+/+}$ and *Ide*$^{-/-}$ islets.

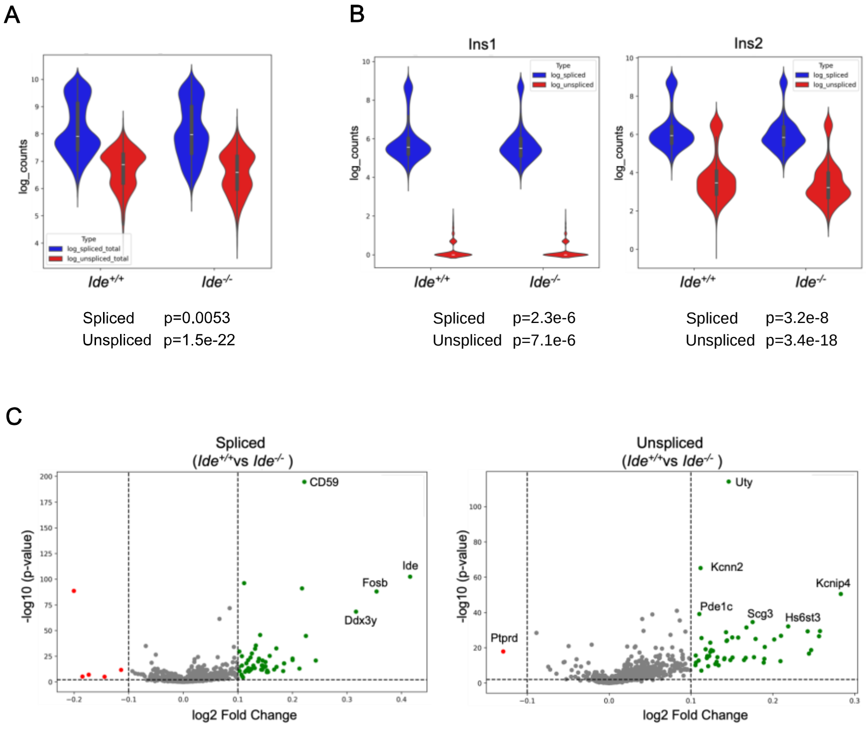

Figure S5. Differential transcript splicing in *Ide*$^{+/+}$ versus *Ide*$^{-/-}$ islet cells, analyzed in single-cell RNAseq data. a) Comparison of the frequency of spliced and unspliced transcripts in the global transcriptome of *Ide*$^{+/+}$ versus *Ide*$^{-/-}$ islet cells. Unspliced transcripts were more frequent in Ide$^{+/+}$ cells. b) Comparison of the frequency of spliced and unspliced *Ins1* and *Ins2* transcripts in the global transcriptome of *Ide*$^{+/+}$ versus *Ide*$^{-/-}$ islet cells. Spliced *Ins1* and *Ins2* transcripts were more abundant in *Ide*$^{+/+}$ cells while unspliced *Ins2* transcripts were more frequent in *Ide*$^{+/+}$ cells. c) Scatterplots of individual spliced and unspliced enriched in *Ide*$^{+/+}$ versus *Ide*$^{-/-}$.

Table S1: Primers for Ide cloning.

Primers	
Cyto-IDE XhoI forward	' GAAGT**CTCGAG**GCCACCATGAATAATCCAGCCATCAAGAG
IDE EcoRI reverse	' CCACGCTT**GAATTC**GGTCATG
V5 Tag XhoI Forward	' GAAGT**CTCGAG**GCCACCATGGGCAAGCCCATCCCCAACCCCCT
TurboID MluI Reverse	' GCCAGA**ACGCGT**CCCGTCCAG

Table S2: Reagents and Resources used.

**Reagent or Resource**	**Source**	**Identifier**
**Antibody**		
Anti-IDE (polyclonal)	Abcam	ab32216
Anti-IDE (monoclonal)	Santa Cruz Biotechnology	sc-393887
V5 Tag (monoclonal)	Invitrogen	R96025
TOM20 (polyclonal)	Proteintech	11802-1-AP
Anti-rabbit IgG, HRP linked	Cell signaling Technology	7074
Anti-mouse IgG, HRP linked	Cell signaling Technology	7076
Anti-CNOT2 (Rabbit polyclonal)	Proteintech	10313-1-AP
Anti-CNOT3 (Rabbit polyclonal)	Proteintech	10335-1-AP
Anti-CNOT8 (Rabbit polyclonal)	Proteintech	10752-1-AP
**Chemicals**		
NucleoBond Xtra Midi EF	Macherey-Nagel	740420.10
DMEM	Thermo Fisher Scientific	1965092
HBSS	Thermo Fisher Scientific	13185052
Biotin	Sigma-Aldrich	B4501
Complete EDTA-free protease inhibitors	Roche Diagnostic	11873580001
NuPage 4-12% Bis-Tris Gel	Thermo Fisher Scientific	NP0322BOX
iBlot2 PVDF Mini Stacks	Thermo Fisher Scientific	IB24002
Streptavidin HRP	BD Biosciences	554066
SuperSignalTM West Pico PLUS Chemiluminescent	ThermoFisher Scientific	34580
BLUeye Prestained Protein Ladder	Sigma	94964
NuPage reducing agent	Thermo Fisher Scientific	NP0004
4x Laemmli sample buffer	BioRad	1610747
VECTASHIELD® PLUS Antifade Mounting Medium	Eurobio Scientific	H-1900-10
Duolink In Situ Wash Buffer	Sigma-Aldrich	DUO82049
Duolink In Situ Detection reagent	Sigma-Aldrich	DUO92014
Duolink PLA anti Mouse Plus	Sigma-Aldrich	DUO92001
Duolink PLA anti Rabbit Minus	Sigma-Aldrich	DUO92005
Collagenase P	Merk (Roche)	11213857001
Accutase	Thermo Fisher Scientific	00-4555
Calcein	Thermo Fisher Scientific	C1430
Draq7	BD Bioscience	564904

AMpure XP	Beckman Coulter	A63880
Qubit dsDNA HS Assay kit	Thermo Fisher Scientific	Q32851
**Software**		
ICY	Icy community platform	https://icy.bioimageanalysis.org
ImageJ	ImageJ	https://imagej.net/ij/.
GraphPadv10		
Metascape	Metascape	https://metascape.org
GSEA	GSEA	https://gsea-msigdb.org
Affinity designer 2	Serif Ltd	https://affinity.serif.com
**Deposited Data**		
Proteomics data	This study	
Single Cell RNA-seq Data	This study	
